# A Review of HIV Pre-exposure Prophylaxis Streamlining Strategies

**DOI:** 10.1007/s11904-020-00528-9

**Published:** 2020-09-13

**Authors:** Aaron J. Siegler, Kevin Steehler, Jessica M. Sales, Douglas S. Krakower

**Affiliations:** 1 Department of Behavioral Sciences and Health Education, Emory University, Rollins School of Public Health, Atlanta, GA USA; 2 Emory University, School of Medicine, Atlanta, GA 30322 USA; 3 Division of Infectious Diseases, Beth Israel Deaconess Medical Center, The Fenway Institute, Fenway Health, Department of Population Medicine, Harvard Medical School, Boston, MA USA

**Keywords:** Chronic care model, mHealth, Telemedicine, PrEP

## Abstract

**Purpose of Review:**

Standard care for HIV pre-exposure prophylaxis (PrEP) in the USA creates substantial burdens for patients, clinicians, and the healthcare system; to optimize uptake, there is a need for innovative strategies to streamline its provision.

**Recent Findings:**

Our review, structured by the expanded chronic care model, identified eleven promising strategies to streamline PrEP care. Approaches ranged widely in mechanism of action. Using text messages to support care was the only strategy with clinical trial evidence supporting its use. Other modalities such as patient navigation, telemedicine PrEP models, alternate dosing availability, same-day prescription, and provider training have promising pilot or associational data and seem likely to lower barriers to entering into or remaining in care. Many of the strategies have established success in related domains such as HIV care, meriting consideration in evaluating their use for PrEP.

**Summary:**

Making PrEP care less burdensome will be an important part of bringing it to scale. Text message interventions have proven efficacy and merit broad adoption. Encouraging preliminary evidence for other strategies indicates the importance of building a stronger evidence base to clarify the effect of each strategy. Ongoing development of an evidence base should not delay the use of these promising strategies; instead, it calls for careful consideration for how each program may best match its environment to facilitate PrEP prescribing and use.

## Introduction

The first major trial of HIV pre-exposure prophylaxis (PrEP), iPrEX, demonstrated the high efficacy of PrEP in 2010 [1]. By 2018, an estimated 380,000 persons had initiated PrEP globally, with the majority (225,000) residing in the USA [2]. In New South Wales, Australia, an encouraging PrEP implementation project initiated 9700 PrEP users across 21 sites. A population-level decline in new HIV diagnoses was observed after the first phase of the project, with 295 new diagnoses decreasing to 221 new diagnoses, a 25% decline. Similarly, in San Francisco, as PrEP and other “getting to zero” efforts scaled up rapidly, new HIV diagnoses were observed to decline substantially [3]. To observe similar population-level gains elsewhere, it will be necessary to bring PrEP to scale for groups with high HIV incidence. Scaling up PrEP to optimal levels will require a considerable investment and effort. UNAIDS has set a goal of having 3 million PrEP users by 2020, and current progress is one-tenth of that level.

PrEP is a biobehavioral intervention requiring clinician oversight of care. Nearly all guidance for PrEP care, including from the World Health Organization and from the US Centers for Disease Control and Prevention [4, 5], places substantial responsibilities on both individuals seeking PrEP and healthcare systems providing it. For individuals, seeking care typically comprises four clinical visits per year with laboratory testing, counseling as needed, and renewed prescriptions. For healthcare systems, PrEP visits require both clinician time and physical resources to host the visit, including laboratory services and supplies. The overall model of PrEP care therefore requires training providers to prescribe PrEP, developing systems to support the financing of PrEP medication, and supporting the quarterly monitoring of individuals over time to ensure they can remain on PrEP.

In order to expand access to the millions in need, it will be important to ease burdens for PrEP users, prescribing clinicians, and healthcare systems. In this article, we review advances to date in efforts to streamline PrEP care, with a focus on the healthcare system of the USA.

## Expanded Chronic Care Model

**Fig. 1:**
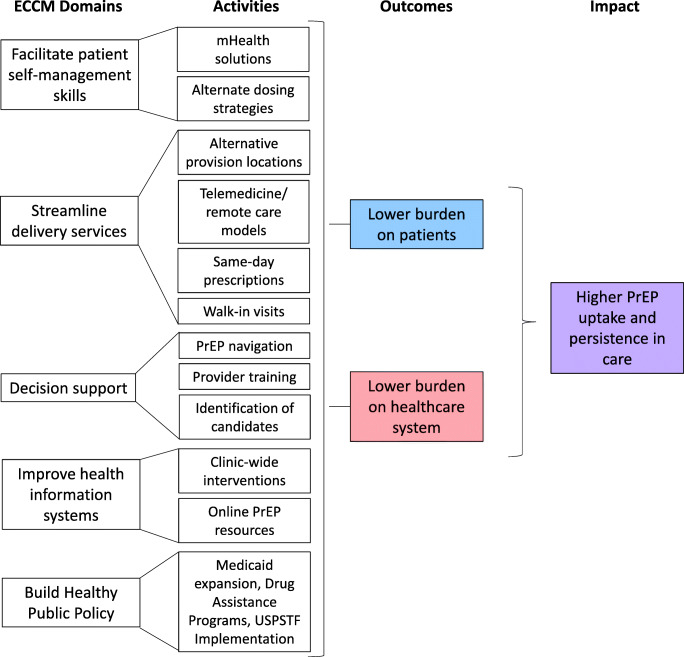
Chronic care model applied to simplification of PrEP care

We leverage components of the expanded chronic care model (ECCM) to structure our review of opportunities for streamlining PrEP care, focusing on interventions that decrease burdens borne by patients and healthcare systems (Fig. 1). The chronic care model was developed to facilitate the implementation of evidence-based methods into disease management practices and has since been expanded to incorporate prevention practice to allow for its integration into public health [6]. ECCM has been widely used and has a strong base of evidence with both randomized controlled trials and implementation science designs indicating efficacy across a variety of health conditions [7]. We selected ECCM domains that align with our review of current evidence regarding simplified PrEP care [6, 8]. For clinicians and community members to optimize decisions to engage in care (decision support), they need a lower burden of care (streamlined delivery approaches), self-efficacy to engage and continue in care (patient skill management), systems to support their preventive health choices (improved health information systems), and healthy public policies to support all components of the model. We review interventions in each area, describing current research and future opportunities.

### Facilitate Patient Management Support

#### mHealth Solutions

There are numerous mHealth-based PrEP adherence and retention support interventions. At their most basic, interventions provide electronic reminders to take daily PrEP through means such as electronic text messages (SMS) or in-app notifications. Weekly text message reminder systems have been demonstrated in international settings to improve adherence to HIV medication regimens [9]. The proliferation of free pill reminder smartphone apps, as well as native, customizable reminder alerts/alarms in smartphone operating systems, makes such trials harder to assess in settings with higher smartphone ownership.

Two trials among MSM in the USA sought to increase PrEP adherence through enhanced messaging formats. The TAPIR trial assessed provision of automated, personalized daily PrEP text message reminders, finding no improvement in the primary outcome of protective levels of PrEP as determined by levels of tenofovir-diphosphate (TFV-DP) indicative of ≥ 4 days per week dosing [10]. A secondary analysis found a TFV-DP threshold indicative of 7 days per week dosing that was higher in the intervention than the control condition, signaling that the intervention may have had a larger impact on a population with lower overall PrEP adherence. The EPIC trial assessed provision of an interactive text message platform with weekly “check-in” messages from study counselors and optional automated daily PrEP reminders [11••]. This intervention had a significant impact on retention in care with 86% of intervention versus 71% of control participants completing all visits. Intervention participants also had higher adherence (72%) than control participants (57%) based on TFV-DP levels indicating ≥ 4 days per week dosing. The TAPIR and EPIC trials differed both by intervention type (automated versus interactive texting) and control patient adherence levels (high versus moderate), so differences in study outcomes may be due to either or both of these factors. These results merit further exploration, possibly in an implementation science framework to explore how to best scale up these promising interventions.

App and mobile-optimized web approaches are promising avenues for supporting PrEP initiation, although no clinical trial data are currently available regarding their efficacy. One pilot study, HealthMindr, developed an app with a diverse array of HIV prevention services: screening for PrEP and post-exposure prophylaxis; prevention recommendations; no-cost prevention mail kits for condoms, lubricant, and HIV self-tests; and prevention locator services [12]. During the four-month follow-up, a majority of study participants ordered condoms and HIV tests, and 9% (8/86) initiated PrEP. A number of clinical trials that use mHealth interventions to promote PrEP initiation or retention in care are underway. One trial adapts HealthMindr to youth populations, using social cognitive theory and substantial formative work [13], and another based on the information, motivation, and behavioral skills’ theory uses personalized risk scores, electronic diaries, and home testing to promote PrEP uptake [14]. A third trial focuses on retention in PrEP care by building on an app platform featuring social network and game-based components [15]. As results from these trials become available, it may be more clear which theoretical models and app services are best suited for supporting PrEP initiation and adherence.

#### Alternate Dosing Strategies

Daily PrEP dosing strategies are the only regimens with US Food and Drug Administration approval, but the World Health Organization, as well as health departments in New York City and San Francisco, have endorsed event-driven (ED) PrEP for MSM. This strategy has been covered in substantial depth elsewhere [16–21]. In brief, ED PrEP has most commonly been 4 pills of tenofovir disoproxil fumarate and emtricitabine (TDF/FTC): 2 pills 2–24 h before sex, 1 pill 24 h after the first dose, and 1 pill 48 h after the first dose (2-1-1). In the IPERGAY trial among MSM in France, as well as several subsequent open-label studies in Europe, ED PrEP performed exceptionally well, with an efficacy similar to daily PrEP [16–18]. ED PrEP performed less well in terms of adherence in HPTN067 among predominantly Black men at the Harlem site, with fewer sexual events covered with medication doses [22]. Modeling from these data indicates that scaling ED PrEP in lieu of daily PrEP might result in higher HIV transmission, particularly for minority MSM [23]. HPTN067 was conducted before the efficacy of event-driven PrEP was fully understood, though, which may limit the generalizability of these modeling results. Recently, no new HIV infections in 136 person years of follow-up were observed among a cohort of predominantly White and Asian ED PrEP users with Kaiser Permanente Insurance, with few reporting side effects or missed doses [24]. The eventual impact of ED PrEP scale-up, however, will depend on (1) the extent to which a clinician-recommended ED strategy could encourage PrEP use among persons who would otherwise not take it, (2) the proportion of daily users that might switch to a ED strategy, and (3) whether the ED strategy over the long-term would increase or decrease prevention-effective adherence as measured by coverage of HIV exposures. ED strategies have the potential to alleviate high rates of PrEP discontinuation among men; one national study in the USA found that only 56% of daily PrEP users persisted through 1 year of PrEP use and only 41% persisted through 2 years of use. Clinical studies have demonstrated that ED PrEP is efficacious when men are adherent to the dosing schedule, so it should receive substantial consideration as an option to facilitate population-level PrEP scale-up among MSM. It is important to note that this strategy has not yet been tested in women and that there are concerns that ED PrEP might be less efficacious in women or transgender persons receiving gender-affirming hormone therapy because of possible sex differences in the pharmacology of tenofovir-based PrEP.

### Streamline Delivery Services

#### Alternative Provision Locations

PrEP is most often provided by clinicians in standard clinic settings. One concept to improve the patient experience is to prescribe PrEP from alternative sites. This has several potential advantages: (1) alternative sites may be more conveniently located compared with centralized clinics/hospitals, (2) some individuals may feel more comfortable seeking preventive care outside of a standard clinic visit, and (3) increased visibility of alternative sites may spur uptake of an otherwise unplanned behavior. These advantages are similar to those gained by HIV mobile testing units relative to clinic-based testing. Mobile HIV testing was found in one clinical trial to increase HIV testing by over 4 times [25] and has been observed to increase participation of important subpopulations such as first-time testers [26]. Encouragingly, PrEP provision through a mobile clinic such as a van is a feasible strategy for outreach to low-service areas; conducted among a predominantly Hispanic population, 168/225 mobile clinic clients sought PrEP and the majority sought a follow-up visit within 3 months [27]. Other promising venues are public health programs, community health centers, STI/HIV testing clinics, and other locations where at-risk persons receive care [28–30]. Each of these may offer unique opportunities to engage persons in considering PrEP care, which is especially important in light of the evidence that repeated offers of PrEP over time can facilitate PrEP initiation [31, 32].

Several studies have used collaborative practice agreements to allow pharmacists to prescribe PrEP from their pharmacies [33–35]. All studies found the programs to be highly acceptable and feasible, but among the two with follow-up data, one found high retention in care, [34] and the other found lower retention in care (28% at 12 months) [33]. Also, mixed results were found for the ability to recruit a diverse patient population, with one having notable success in recruiting Latino MSM [35•] and other studies not reporting or having predominantly White patient populations. Given legal regulations regarding collaborative practice agreements, such programs may not be scalable unless states change their laws regarding PrEP. Recently, California made just such a change, enacting California Senate Bill 159 that would allow pharmacists to prescribe to individuals a single, 60-day regimen of PrEP, which could lower the barrier of initiating PrEP [36].

Another model is for providers at acute or retail care clinics, often housed within pharmacies, to prescribe PrEP. Such clinics offer on-demand appointments that do not require scheduling or expectation of follow-up [37, 38]. Two pharmacy chains in the USA, Walgreens and CVS, have implemented PrEP care nationally in their retail care clinics, and the organizations have agreed to have their clinics listed in a national database of PrEP-prescribing clinics [39]. A literature review comparing the efficacy of retail care clinics vs. physician offices for chronic disease management revealed a lack of evidence-based data favoring either setting [37]. An additional literature review comparing retail care clinics with other sites of care in terms of cost, quality, and patient satisfaction found inconsistent measures across a total of 15 studies that greatly limited the ability to make definitive conclusions [37, 38]. The reviews note the growing popularity of such clinics, yet indicate there is insufficient evidence to inform patients, clinicians, and policymakers in expanding the use of this important resource [37, 38].

#### Telemedicine/Remote Care Models

One step beyond alternative PrEP care sites is telemedicine, which could make in-person medical visits non-obligatory. There are a number of substantial potential benefits of remote care approaches for PrEP, including reduced transit time, effort, and cost; reduced potential stigma in seeking care; and lower burdens on busy clinical sites. Perhaps the most compelling is that having four in-person clinic visits each year specifically for using medications with an excellent safety profile could be unnecessary and unsustainable for many. Such burdens are not imposed for other well-tolerated medications such as oral contraceptives or statins, likely because it could adversely impact retention. When provided the hypothetical choice, the majority of participants in a national sample of MSM preferred home-based PrEP care to clinic-based care [40]. Two small pilot studies of telemedicine PrEP indicate feasibility and promise of the approach, although the small sample for each study limits larger conclusions [41, 42]. A randomized trial of telemedicine PrEP, using an app-based platform to facilitate the remote care, is currently underway and should provide more definitive evidence regarding the impact of telemedicine PrEP on initiation and maintenance in care [43]. To optimize the likelihood of impact, all aspects of PrEP care in the trial can be completed from home, including self-collection of specimens for laboratory testing and mailing of prescriptions when possible. A number of commercial telemedicine PrEP companies have emerged. For individuals who are underinsured, uninsured, or use Medicare/Medicaid, payment barriers may exist to accessing such commercial services, although some note on their websites that payment assistance navigation is provided.

A different model of remote PrEP care seeks to reduce the number of required visits. For instance, a single annual in-person visit could be with a clinician, and three intervening visits could be performed by remote lab testing and an electronic survey. A pilot test of this model, conducted by the study authors, found that most participants preferred it to standard care and that 40% would be more willing to remain on PrEP if this option was provided [44]. A challenge for both full telemedicine PrEP and alternative reduced burden models is payment and billing. Addressing such issues could be accomplished with legislative or other systems-based changes and would facilitate implementation of telemedicine PrEP approaches.

#### Same-Day Prescriptions

Same-day prescription refers to PrEP initiation on the same day as a patient’s first visit with a PrEP provider. Many clinicians currently use a referral model, with PrEP discussion and evaluation followed by either a referral to another clinic or a scheduled follow-up appointment for PrEP initiation after laboratory testing is complete. Referral models can introduce several days or more of delay between initial patient contact and PrEP initiation—with an inherent potential for loss to follow-up (i.e., primary non-adherence). For HIV treatment initiation, same-day prescriptions after positive testing have had success [45, 46]. Denver Public Health has constructed a model for same-day PrEP at walk-in STI clinics, including PrEP eligibility assessment, serum creatinine testing, hepatitis B testing, urine pregnancy testing, point-of-care HIV testing, patient navigator counseling, and 30-day PrEP prescription; when this system was used, 78% of patients returned for a 1-month follow-up visit, and 57% returned for a follow-up visit at 3 months [47]. A New York City study that used medical chart review found that same-day initiation of PrEP was associated with decreased retention in care at the third follow-up visit [48]. The authors did not speculate on the reasons for this unexpected association. It is possible that more non-adherent patients were classified as same-day initiators relative to patients in the delay-care group (e.g., patients in the delay care group may have discussed PrEP with their clinician but never returned for a formal “PrEP initiation” visit, and therefore were counted as not starting rather than discontinuing). Thus, it is challenging to determine the overall impact of a same-day program without additional information to supplement a medical records’ review. A study in Kenya developed an approach for same-day PrEP among pregnant and postpartum women, with assessment ongoing [49]. Additional studies are needed to determine the benefits and potential limitations of same-day PrEP programs.

#### Walk-In Visits

Scheduling of a healthcare visit for persons who are frequently healthy and young, and therefore potentially unaccustomed to scheduling regular clinician visits, may be a barrier to getting PrEP. Many STI and HIV testing sites already exist as walk-in clinics to address this challenge. These anonymous clinics do not require scheduled visits during regular hours, and these sites are often preferred among key populations such as men who have sex with men (MSM) and persons who engage in sex work [50]. A survey at the Harris Health System in Houston Texas found that individuals ≥ 18 years old presenting for walk-in HIV testing expressed interest in taking PrEP at a rate of about 73% [51], suggesting an opportunity to engage patients in PrEP after walk-in HIV testing. PrEP studies conducted by Denver Public Health utilized walk-in visits for PrEP initiation [47]; however, no comparisons exist with appointment-based models. Moreover, subsequent PrEP care follow-up visits are scheduled months in advance, potentially easing the challenge of visit scheduling. Further assessments of walk-in visit models for PrEP care are needed, given demonstrated interest among key populations and limited data for its application to sexual health.

### Decision Support

#### PrEP Navigation

In general, patient navigation is defined as service provided by non-clinical staff members to assist patients in addressing barriers to PrEP care [52]. PrEP navigation initiatives are often comprised of trusted peers who possess shared lived experiences with the community and are frequently adopted and supported by clinics with large numbers of PrEP patients. [52, 53] These services can also be used to offset time burden on clinicians, thereby alleviating a potential barrier to clinician capacity and willingness to prescribing PrEP. Several studies explored PrEP navigation in implementation or pilot study settings, indicating success for various types of PrEP navigation with obtaining higher PrEP adherence or earlier PrEP initiation [54–56]. Studies of PrEP navigation and its utility are ongoing, with a review by Pinto et al. noting at least 6 NIH-funded studies of “PrEP navigation” focusing on “black MSM, young Latino MSM, women upon release from incarceration, people who inject drugs (PWID), and methamphetamine users.” [53] A number of online resources for PrEP navigation may facilitate its adoption into the practice of providers currently prescribing PrEP. For instance, PleasePrEPMe and the NYC Health Department offer training for various methods of PrEP navigation, featuring online courses and detailed manuals of operation [57–59].

#### Provider Training

Despite the Centers for Disease Control and Prevention (CDC) first publishing draft PrEP practice guidelines in 2012 and releasing formal clinical guidelines in 2014, a recent survey of primary care providers (PCPs) identified that although most PCPs were interested in prescribing PrEP, only 17% of PCPs had ever prescribed it, and only 33% had ever discussed it with patients [60, 61]. Reasons identified for low levels of PrEP discussion and prescription included limited knowledge of PrEP and concerns about insurance and other barriers. An additional barrier to PrEP provision by PCPs has been described as a “purview paradox,” in which PCPs generally view PrEP prescription as a responsibility of HIV specialists—whereas most HIV negative patients who are eligible for PrEP do not possess any need for such specialized care [62]. Low PrEP prescribing is not unique to PCPs; a national survey of family planning providers, a group with specialized expertise in sexual health services, found low PrEP knowledge and use overall among this group [63]. Only one-third of respondents could correctly define PrEP and its efficacy, and less than 5% had ever prescribed PrEP. The majority felt uncomfortable prescribing PrEP due to lack of training.

A literature review of provider educational interventions for increasing PrEP implementation in primary care settings lists suggestions of clinical scenarios that should be included for provider training [64], and qualitative studies provide useful information regarding which modalities for training are preferred among providers [65]. A single 20–60-min in-person educational presentation at 14 Duke PCP offices resulted in a fourfold increase (OR 4.8) in self-reported PrEP prescriptions among physicians who attended the session [66]. Similarly, a 1.5-h in-person educational training at 4 family planning clinics in Metro Atlanta resulted in increased provider PrEP knowledge and confidence to identify women who may benefit from PrEP [67]. Post-training exit interviews with patients revealed that providers discussed HIV risk and PrEP with the majority of women with PrEP indications. Encouragingly, participants in a PrEP-based extension of the Project ECHO (Extension for Community Healthcare Outcomes) telementoring program had improved knowledge and reported increased likelihood of prescribing PrEP two years after their training [68]. Other in-depth PrEP provider training support resources exist, such as an online, continuing medical education course (“PrEParing”) created at Johns Hopkins University that exposes learners to 12 h of content over 6 weeks [69]. For more complex medical issues or questions, the University of California, San Francisco, offers a support line for clinicians with questions about PrEP [70]. Although the most effective timing, content, and modality of PrEP provider education are not yet clear, it is likely that a variety of options may be beneficial to meet individual preferences and knowledge deficits.

#### Identification of PrEP Candidates

Ideally, clinical providers would elicit comprehensive sexual and substance use histories with all of their patients as part of routine healthcare, which would facilitate identification of persons who are likely to benefit from PrEP use. Given competing priorities and intensive time constraints, though, providers do not typically ascertain indications for PrEP for many of their patients.

Several tools have been developed that could help streamline the process by which providers identify candidates for PrEP. Clinical prediction rules have been developed and validated to identify persons at increased risk for future HIV acquisition based on self-reported demographic and behavioral questions, such as age, race, and sexual and substance use behaviors. These tools could be used at the point-of-care to identify people for discussions about PrEP. However, these tools have only been developed for MSM and people who inject drugs (i.e., not for transgender people or cisgender women), they have suboptimal predictive accuracy [71–74], and some have lower sensitivity for specific populations (e.g. black MSM) [75, 76], all of which could limit their utility.

An additional strategy for identifying PrEP candidates is to use routine electronic health records (EHR) data to alert providers about individual patients at increased risk for HIV acquisition. These methods, unlike the prediction tools mentioned above, have the benefit of using existing components of health records rather than screening data. This avoids having clinicians potentially serving as PrEP gate-keepers, potentially democratizing the selection process if implemented appropriately. Studies have used machine learning methodologies to identify individuals at increased risk for incident HIV in large healthcare systems using EHR data [77, 78]. These automated prediction algorithms have had high predictive performance for identifying men who may benefit from PrEP use in general care settings, with predictive accuracy that is comparable with that of prediction rules commonly used in other areas of preventive medicine (e.g., the Framingham Risk Score) [79]. However, prediction models have had poor performance at identifying women in these settings thus far, which could be in part because the models were trained using data that included very few women with incident HIV. Additional studies to test the impact of implementing EHR prediction models on PrEP use and to improve their performance for women are underway. Moreover, these tools must be implemented carefully and, in awareness of existing disparities by race/ethnicity, ideally explicitly targeted to minimize care access disparities.

### Improve Health Information Systems

#### Clinic-Wide Interventions

Slow adoption of any new evidence-based practice, like PrEP, into clinical care is a widespread concern among healthcare systems [80, 81], with some noting an average 15- to 20-year lag before a new evidence-based practice is widely integrated into routine care [82]. Clinics have difficulty systematically implementing new practices, and often this is due to challenges coordinating change across multiple aspects of a practice setting, rather than their lack of recognizing a new practice as relevant and desirable to provide [80, 83]. PrEP care requires a higher level of engagement of healthcare providers, along with clinic support staff, than has typically been required for HIV prevention (e.g., condom-focused), and has cost and resource considerations relevant for clinics. The steps in PrEP delivery include the following: (1) eliciting a patient’s sexual and substance use history to inform the potential benefits of PrEP use, (2) providing PrEP counseling, (3) assessing a patient’s candidacy for PrEP via labs, (4) prescribing PrEP, and (5) follow-up and clinical monitoring. During Step 1, the patients are screened for HIV risk factors and tested for HIV (noting that HIV testing could also happen during Step 3). During Step 2, the patients are provided with information about PrEP and their interest and potential to adhere to PrEP are assessed. During Step 3, the patients are assessed for any signs and symptoms of undiagnosed HIV infection and receive laboratory testing for kidney function, hepatitis B and C, pregnancy (when relevant), and other STIs. Step 4 involves prescribing PrEP to patients who meet clinical criteria and express interest. This step also may involve enrolling the patient in insurance or medication assistance programs to ensure they can pay for PrEP, which can involve intensive health navigation for some patients. Step 5 involves follow-up visits every 3 months for HIV testing, adherence counseling, risk reduction support, side effect assessment, pregnancy testing, STI testing, and kidney function testing. Due to differences in organizational structure, services provided, community partnerships, capacity (staffing and financial), and resources, clinics may require varying levels of intervention focused on one or more of the aforementioned steps to support the delivery of onsite PrEP care [84].

Clinic-level interventions consist of varying implementation strategies to enhance the adoption, implementation, and sustainability of a new clinical practice. Powell et al. have characterized implementation strategies into 6 groups: planning (e.g., conducting a clinic-level organizational assessment, developing a formal implementation plan), educating (e.g., conduct educational trainings, distribute educational materials), financing (e.g., access new funding), restructuring (e.g., task-shifting), managing quality (e.g., provide clinical supervision, audit and feedback, EHR or patient reminder systems), and attending to policy context (e.g., creating or changing scope of practice restrictions) [85]. Often a combination of implementation strategies is required to achieve clinic-wide practice change [86]. The majority of studies among providers have concluded that more education about PrEP and its delivery and access to tools (e.g., HIV risk assessment screeners, EHR-based risk algorithms, patient educational resources) are needed to optimize PrEP implementation [62, 87–89]. Even as knowledge about PrEP increases, prescribing providers may still have concerns about how adding PrEP counseling, ordering/reviewing labs, and monitoring would add substantial time to limited visit allowance per patient. Task-shifting models of PrEP care, such as those employing patient navigators to support patient education and financial navigation, have been successfully implemented, even in resource-constrained environments like safety-net community clinics [53–56]. Robust evaluations of PrEP implementation plans that distribute the tasks associated with PrEP (e.g., screening, education, testing, and monitoring) across various clinic staff to offset the burden of PrEP care falling on providers are warranted. Given the multiple steps of PrEP care, it is likely that more than one implementation strategy will be necessary to facilitate scaling PrEP in various clinical or alternative care environments. Encouragingly, innovative combination interventions, like Saberi and colleagues’ program consisting of patient support staff to handle screening and insurance navigation in addition to a web-based management tool to facilitated patient follow-up, have recently been developed [54]. However, there is a need for rigorous evaluations of PrEP implementation interventions, particularly those that combine multiple implementation strategies.

#### Online PrEP Resources

As clinics and the health systems make changes to enhance access to PrEP care, it is important for online resources to similarly facilitate access. One such resource is the community-based Facebook group PrEP Facts [90], which has over 20,000 members and seeks to support persons interested in or on PrEP. The moderated group, founded by Damon L. Jacobs, has participation via request in order to increase privacy and facilitate sharing of accurate information. Another resource is PrEP Locator, a national listing of PrEP providers in the USA that was created by the authors and is now supported by the US Centers for Disease Control and Prevention [39]. The PrEP Locator website has had over one million views and over one-half million users since its launch in late 2016, in spite of no advertising budget for the site. A pilot user study found that most users (48/54, 89%) expected the site to be useful in the future, over one-third reported that the site helped them find a provider (21/54), and over 10% (7/54) had moved forward on the PrEP care cascade by either scheduling or completing a PrEP initiation visit within 1 month of visiting the site [91]. The use and utility of this site indicate the promise of novel and easily accessible online resources. Another freely available online resource, the “What is PrEP” video, which provides a detailed and accessible description of PrEP and PrEP care, has been viewed over 200,000 times according to its website tracker. Given the high interest in and use of these free online resources, future interventions that seek to leverage them to enhance engagement in the PrEP care cascade represent a promising choice worthy of implementation science research.

### Building Healthy Public Policy

Laws and their implementation intersect with PrEP care and services in concrete ways that can facilitate or impede PrEP use [92]. At the local level, some health departments make PrEP referrals, provide education to the public or medical providers, or even prescribe PrEP directly through health department clinics [93]. At the state level, PrEP Drug Assistance Programs (PrEP-DAP) are offered by a number of states and cover medication costs or ancillary costs such as laboratory testing and clinical visit fees. Separately, Medicaid expansion policies have led to increased numbers of persons accessing health insurance coverage [94]. One recent study in the USA found that the states that had either PrEP-DAP or expanded Medicaid had 25% higher PrEP prescriptions per population and that the states that had enacted both policies had 99% higher PrEP prescriptions per population [95]. At the national level, the US Preventive Services Task Force in 2019 rated PrEP with their highest recommendation (Grade A), meaning that there is “high certainty that the net benefit is substantial.” [96] This rating has considerable implications, because it requires that commercial insurers cover the intervention medication cost without any cost sharing. It is currently unclear whether this coverage would also include the ancillary services that are required to receive a PrEP prescription [97].

## Conclusion

Streamlining PrEP care has the potential to improve initiation of and persistence on PrEP, which are the major barriers to PrEP having an optimal population-level impact. The ECCM provided a useful framework to consider the panoply of interventions that could contribute to optimizing PrEP delivery. This review identified a number of promising strategies, including evidence-based interventions. There is a need to further develop an evidence base regarding other PrEP streamlining modalities, with future evidence to be created through future clinical trials or rigorously evaluated implementation science studies. These efforts should include cost-effectiveness assessments to inform future resource allocation strategies. In the meantime, programs should seek to streamline their procedures in accordance with the needs of their clients. Many clinics are currently offering same-day PrEP, using dedicated PrEP navigators, or reducing visit schedules. The laudable goal of these programs is to provide patient-centered care that reduces barriers to PrEP, and we urge that these efforts continue as an evidence base is built to inform which strategies are most efficacious.
